# Polysaccharides from *Trametes versicolor* as a Potential Prebiotic to Improve the Gut Microbiota in High-Fat Diet Mice

**DOI:** 10.3390/microorganisms12081654

**Published:** 2024-08-13

**Authors:** Ming Bai, Zhenfeng Huang, Xiaoya Zheng, Mingyong Hou, Song Zhang

**Affiliations:** School of Life Science, South China Normal University, Guangzhou 510631, China

**Keywords:** *Trametes versicolor*, polysaccharides, intestinal fungi, gut microbiota, hyperlipidemia

## Abstract

Polysaccharides derived from *Trametes versicolor* have been found to exhibit hypolipidemic activity in hyperlipidemic mice, but the mechanism by which they modulate intestinal flora is still unclear. Currently, this study aimed to investigate the regulatory effects of extracellular (EPTV) and intracellular polysaccharides from *T. versicolor* (IPTV) on the dysbiosis of intestinal flora in mice fed a high-fat diet (HFD). The results showed that the oral administration of *T. versicolor* polysaccharides significantly ameliorated lipid accumulation and steatosis in hepatocytes. The gut dysbiosis in the HFD mice was characterized by a decrease in abundance and diversity of bacteria and an increase in the Firmicutes/Bacteroidetes ratio. However, *T. versicolor* polysaccharides attenuated these changes and reduced the relative abundance of bile-salt-hydrolase (BSH)-producing bacteria, such as *Bacillus*, *Enterococcus*, *Bifidobacterium*, and *Lactococcus*. It is noteworthy that *T. versicolor* polysaccharides also restored the disorganization of intestinal fungi in HFD mice, with EPTV treatment leading to a higher relative abundance of Basidiomycota and Ascomycota compared to IPTV. Additionally, *T. versicolor* polysaccharides enhanced the growth of butyrate-producing bacteria via the *buk* and *but* pathways, accompanied by an increase in short-chain fatty acids (SCFAs), especially butyrate. IPTV also increased the expression of G-protein-coupled receptors 41 (GPR41) and 43 (GPR43) by 40.52% and 113.24% each, as compared to 62.42% and 110.28%, respectively, for EPTV. It is suggested that IPTV and EPTV have the potential to counteract hyperlipidemia-associated intestinal flora disorders and improve lipid metabolism.

## 1. Introduction

Hyperlipidemia is a metabolic disorder characterized by elevated levels of lipids and lipoproteins due to abnormalities in fat metabolism, which is typically attributed to factors such as unbalanced diets, obesity, and hereditary diseases. Over time, as people’s living standards improve and dietary structures change, the incidence of hyperlipidemia has increased, even among younger individuals [1]. This trend has had a detrimental impact on the global health system. Notably, hyperlipidemia is a major risk factor for cardiovascular disease (CVD), which continues to be the leading cause of morbidity and mortality worldwide [2]. Additionally, hyperlipidemia is closely associated with diabetes mellitus, pancreatitis, and neurodegenerative diseases [3,4,5]. Previous studies have shown that hyperlipidemia is characterized by an imbalance in blood cholesterol levels, which is clinically manifested by elevated total cholesterol (TC), low-density lipoprotein cholesterol (LDL-C), triglycerides (TG), and decreased high-density lipoprotein cholesterol (HDL-C). Lowering blood lipid levels is crucial for improving lipid profiles. Currently, the primary treatment for hyperlipidemia involves the use of statins or fibrates as the first-line therapeutic approach. However, adverse effects are still noticeably occurring, particularly affecting the muscles, gastrointestinal tract, liver, and kidneys [6,7]. Therefore, it is of practical significance and application value to develop novel functional foods that exhibit substantial lipid-lowering activity.

In recent years, thanks to excellent nutritional and therapeutic properties, mushrooms have received continuous attention in the field of medicine and food processing. One such mushroom is *Trametes versicolor*, also known as Turkey Tail, which is commonly found in the northern hemisphere. Its medicinal value can be traced back as far as 2000 years ago [8]. *T. versicolor* has long been recognized for its medicinal properties, as it can produce various biologically active metabolites. Natural products derived from *T. versicolor*, including sterols, triterpenoids and polyphenols, have demonstrated encouraging functions [9]. Although *T. versicolor* metabolites are chemically diverse, most pharmacological studies have focused on bioactive polysaccharides. Polysaccharides are a type of macromolecular carbohydrate found in nature, known for their functional diversity. Numerous studies have reported the potential of *T. versicolor* in improving symptoms associated with dyslipidemia, diabetes, and CVD [10]. In a study conducted by Nikolic et al., it was demonstrated that the polysaccharides derived from *T. versicolor* were capable of ameliorating oxidative stress, regulating lipid levels, and providing cardioprotective effects [11]. Moreover, the polysaccharide extract from *T. versicolor* was found to significantly reduce myocardial fibrosis and enhance the cardiovascular system in diabetic rats by modulating the transforming growth factor β1 (TGF-β1)/Smad signaling pathway [12].

Intestinal flora are widely recognized as a crucial factor in maintaining health, as they regulate energy acquisition and the immune system of the host [13]. Increasing evidence has revealed that disturbances in the intestinal flora are associated with the occurrence and progression of various diseases, including obesity [14], immune system disorders [15], and Alzheimer’s disease [16]. It is worth noting that fungi, although present in smaller quantities, also play a significant role in the composition of the intestinal flora. Previous research has highlighted the importance of the intestinal flora, including fungi, in regulating lipid metabolism in the host [17]. However, the composition of intestinal flora is mainly influenced by host diet, genetics, immunity, and age [18]. Among these factors, dietary patterns and different dietary components directly impact the composition of gut flora. Fungal polysaccharides serve as an important dietary source that can modify the composition of intestinal flora and promote the growth of beneficial bacteria. Therefore, it is essential to identify beneficial dietary ingredients that can lower lipid levels by altering the gut flora composition. In our previous study, we found that *T. versicolor* polysaccharides (PTVs) were able to improve dyslipidemia induced by a high-fat diet (HFD) [19], but the underlying mechanism behind the effects of polysaccharides produced by liquid fermentation of *T. versicolor* on both hyperlipidemia and the intestinal flora remains unclear.

Hence, the objective of the present study was to evaluate the potential of supplementing with *T. versicolor* polysaccharides to improve the intestinal bacterial and fungal communities in mice with lipid metabolism disorders. Furthermore, the study aimed to investigate the correlation between these changes and lipid-metabolism-related parameters. The findings of this research can contribute to the development of lipid-lowering medications or functional foods that target the intestinal flora using *T. versicolor* polysaccharides.

## 2. Materials and Methods

### 2.1. Chemicals and Reagents

Simvastatin tablets (Approval number: J20180007) were purchased from Hangzhou Merck Sharp & Dohme Co., Ltd. (Hangzhou, China). The animal diets including a standard diet and an HFD were purchased from Guangdong Medical Laboratory Animal Center (Guangzhou, China), certified No. SCXK (Yue) 2013-0002. All other chemical reagents used were of analytical grade.

### 2.2. Preparation of Polysaccharides

The extracellular (EPTV) and intracellular polysaccharides of *T. versicolor* (IPTV) used for experiment were prepared as previously described [19]. Briefly, *T. versicolor* liquid fermentation products were first centrifuged to separate the supernatant and mycelium. The mycelium was then dried, pulverized, and defatted. It was subsequently extracted in distilled water (75 °C, 1:2 *w*/*v*) for 2.5 h, repeated three times. The supernatant and aqueous extract were concentrated, and the proteins were sequentially removed using trypsin and Sevage methods [20]. Next, 95% ethanol (*v*/*v*) was added to the concentrate, which was left to precipitate overnight at 4 °C. The resulting precipitate was collected through centrifugation. After dialysis and freeze drying, the EPTV and IPTV were obtained. Relevant chemical characterization of polysaccharides has been reported in our previous work. The molecular weight of EPTV is 68.4 kDa, and its monosaccharide constituents are as follows: ribose (0.24%), rhamnose (0.27%), arabinose (0.71%), xylose (7.77%), galactose (19.99%), glucose (34.15%), and mannose (36.87%). IPTV has a molecular weight of 127.0 kDa, and its monosaccharide constituents are as follows: ribose (0.19%), rhamnose (0.22%), arabinose (0.38%), xylose (1.56%), galactose (14.00%), mannose (25.98%), and glucose (57.67%) [19]. 

### 2.3. Animal Experiment

All experimental procedures were conducted following the approved procedures by the Ethics Committee for animal research at South China Normal University, with the approval code SCNU-SLS-2024-026. The procedures were also in accordance with the principles outlined in the National Institutes of Health guide for the Care and Use of Laboratory Animals. Ninety specific-pathogen-free male adult Kunming mice (No. SCXK (Yue) 2018-0002), weighing 16–20 g, were obtained from Guangdong Medical Laboratory Animal Center (Guangzhou, China). The mice were housed in polycarbonate cages, maintained under standardized environmental conditions (55 ± 5% relative humidity, 22 ± 2 °C temperature and 12 h light/dark cycle), and provided with food and water ad libitum throughout the experiment. The HFD used in the study was composed of 1.0% (*w*/*w*) cholesterol, 10% lard, 0.3% sodium cholate, and 88.7% standard diet. Specific dietary formulas are shown in Table S1. 

**Figure 1:**
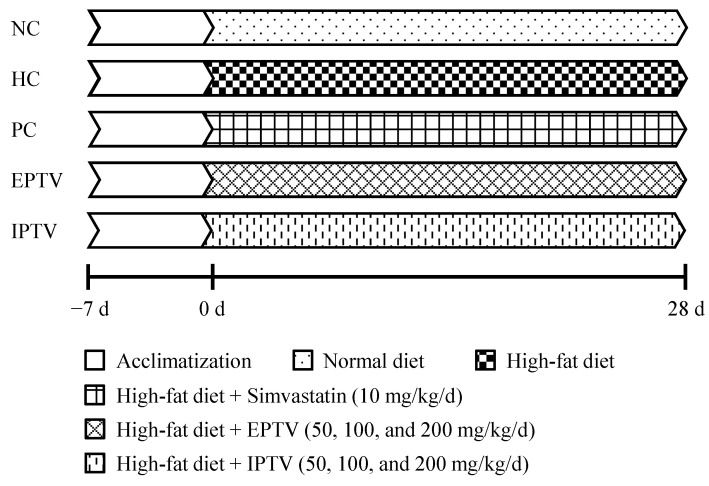
The procedure of animal experiment.

The animal experiment procedure is shown in Figure 1. After a week of acclimation, the mice were randomly divided into 9 groups (*n* = 10 per group) including normal control (NC) group, hyperlipidemic control (HC) group, positive control (PC) group, three dosage groups of IPTV (50, 100, and 200 mg/kg/d), and three dosage groups of EPTV (50, 100, and 200 mg/kg/d). During the experiment, the NC group received a standard diet and was orally administered the same volume of physiological saline. The HC group received an HFD and was intragastrically administered the same volume of physiological saline. The PC group was subjected to both HFD and simvastatin oral treatment, with a simvastatin dosage of 10 mg/kg/d. As a lipid-lowering agent, simvastatin has been proven effective in combating obesity in mice fed an HFD [21]. All other groups consumed HFDs and were given corresponding doses of polysaccharides by gavage. The experiment lasted for 28 consecutive days. 

Behavioral activity, water, and food intake of the mice were observed daily. Body weight was recorded at weekly intervals and their intake was adjusted until the end of the experiment. After a 4-week treatment, all mice were fasted overnight and anesthetized with sodium thiopental before being euthanized by clavicle dislocation. Blood samples were collected and centrifuged at 4 °C and 3000 rpm for 15 min, and the resulting serum was stored at −80 °C until biochemical analysis. After blood collection, the heart, liver, kidney, and spleen were immediately removed, rinsed with ice physiological saline, and weighed. The organ indexes were calculated as the ratio of organ weight to body weight. Colon tissue was homogenized with cold physiological saline, and 10% of tissue homogenate (*w*/*v*) was centrifuged at 4000 rpm for 10 min at 4 °C. The resultant supernatant obtained after centrifugation twice was stored at −20 °C until assay. Stool samples were collected and immediately stored at −80 °C until DNA extraction.

### 2.4. Biochemical Analyses

Serum lipid levels, including TC, TG, and LDL-C, were measured using enzymatic colorimetric methods. Commercial kits from Nanjing JianCheng Institute of Bioengineering (Nanjing, China) were utilized for the measurements. Protein expression of colonic GPR41 and GPR43 was determined using an ELISA kit from Wuhan ColorfulGene Biological Technology Co., Ltd. (Wuhan, China), following the standard procedure. 

### 2.5. Histological Evaluation

At the end of the study, liver tissues were collected and fixed in 4% paraformaldehyde overnight. Subsequently, they were washed with dd H_2_O, rehydrated using gradient ethanol solutions, embedded in paraffin, and cut into 5 µm sections. These sections were then stained with hematoxylin and eosin (H&E) according to a standard method. Histopathological changes were observed and photographed under a microscope (Olympus, Tokyo, Japan) equipped with a digital camera (Olympus, Tokyo, Japan) at a magnification of 400×.

### 2.6. Stool Sampling, DNA Extraction and Sequencing

Microbial genomic DNA was extracted from fecal samples using the bacterial DNA Kit (Omega, Shanghai, China). The DNA concentration was determined by means of a Nanodrop 1000 (Thermo Fisher Scientific, Wilmington, DE, USA) and stored at −20 °C until further processing. The taxonomic composition of bacterial and fungal communities was analyzed by amplifying the V3-V4 hypervariable region of the bacterial 16S rRNA gene and ITS1 of the fungal 18S rRNA gene. This was conducted through a two-step amplification procedure using universal primers pairs 38F (5′-ACTCCTACGGGAGGCAGCAG-3′) and 806R (5′-GGACTACHVGGGTWTCTAAT-3′), ITS1F (5′-CTTGGTCATTTAGAGGAAGTAA-3′) and ITS1R (5′-GCTGCGTTCTTCATCGATGC-3′), which incorporate Illumina adapters and barcode sequences. The DNA extraction, polymerase chain reaction (PCR), and Illumina MiSeq platform (Illumina, San Diego, CA, USA) were carried out at Biomarker Technologies Co., Ltd. (Beijing, China).

### 2.7. Bioinformatics Analysis

The raw reads were quality filtered and merged using Usearch (version 7.1). Then, the sequence reads were classified into operational taxonomic units (OTUs) based on the Ribosomal Database Project (RDP) and UPARSE with a 97% similarity cutoff. The taxonomy analysis was performed on Illumina’s BaseSpace cloud computing platform. OTU abundance data were normalized using a standard sequence number corresponding to the sample with the least sequences. The relative proportion of each OTU was examined at the phylum, class, order, family, genus, and species levels. Alpha (within a community) and beta (between communities) diversity were analyzed with QIIME version 1.7.0. For alpha diversity, Chao1 and Shannon indices were calculated to assess community richness and diversity, respectively. For beta diversity, two-dimensional principal coordination analysis (PCoA) and nonmetric multidimensional scaling (NMDS) plots were generated using the SIMCA-14.1 software (UMETRICS, Umea, Sweden) based on the relative abundance of gut microbiota at the OTU level (Weighted UniFrac). In addition, taxonomic changes that differed significantly between the different groups were analyzed by the linear discriminant analysis (LDA) effect size (LEfSe) algorithm with an alpha value of 0.05 and an LDA score threshold of 4.0 using the LEfSe Tools.

### 2.8. Short-Chain Fatty Acid (SCFA) Analysis

Gas chromatography coupled to mass spectrometry (GC–MS) was used to analyze the contents of SCFAs, including acetic acid, propionic acid, isobutyric acid, n-butyrate, isovaleric acid, and n-valeric acid, in stool samples. At the end of the study, stool samples were collected and immediately stored at −80 °C. To extract SCFAs, 50 mg of stool samples were homogenized in 500 μL of saturated NaCl solution and then acidified with 40 μL of 10% sulfuric acid. After that, 1 mL of diethyl ether was added to the samples, followed by centrifugation at 14,000× *g*, 4 °C for 15 min, and the supernatant was used for GC–MS analysis.

### 2.9. Statistical Analysis

The data were expressed as means ± standard deviation (SD), and determinations were obtained from ten animals per group. To detect significant differences between any two groups, the one-way analysis of variance (ANOVA) followed by Student’s *t*-test was used. *p*-values < 0.05 were considered statistically significant. The correlation network between lipid-metabolic-disorder-related indexes and the intestinal microbiota was plotted using Cytoscape software (Version 3.6.1).

## 3. Results and Discussion

### 3.1. PTV Administration Normalized Body Weight Gain and Organ Indexes in HFD-Fed Mice

A significant increase in body weight was observed in mice after 14 days of high-fat diet treatment (*p* < 0.05, Table S2). However, mice in the IPTV and EPTV groups tended to have slightly lower levels than those in the HC group, although the difference was not statistically significant (*p* > 0.05). Mice supplemented with PTVs displayed a suppressive effect on body weight gain and liver index (*p* < 0.05). The weights of the heart and spleen were similar in all groups, and in agreement with the findings of Sheng et al. [22], the splenic index of obese mice usually showed a slight change without a significant difference (*p* ≥ 0.05).

### 3.2. PTVs Alleviated Dyslipidemia in HFD-Fed Mice

**Figure 2:**
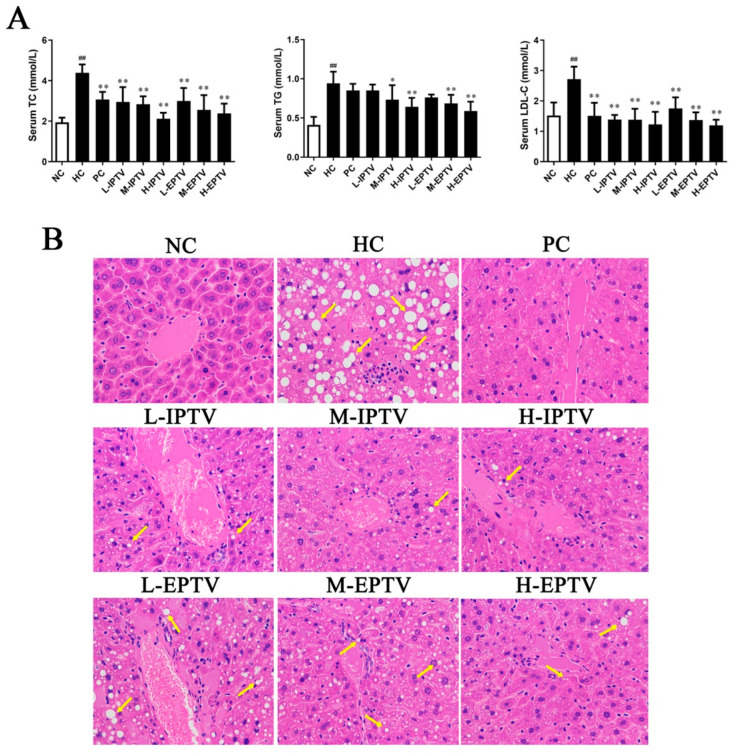
Effects of PTVs on lipid profiles of high-fat-diet-fed mice. (**A**) Serum lipid profiles including total cholesterol (TC), triglyceride (TG), low-density lipoprotein cholesterol (LDL-C). Data are presented as means ± SD, *n* = 10. ^##^ *p* < 0.01, compared with NC group; * *p* < 0.05 and ** *p* < 0.01, compared with HC group. (**B**) HE staining of liver was performed to observe the morphology of liver (×400). Yellow arrow indicates fat pathological changes.

To investigate the potential role of PTVs in hyperlipidemia, mice were fed an HFD and treated with either saline or polysaccharides via oral gavage for 4 weeks. At the end of the experiment, serum lipid profiles were examined. As shown in Figure 2A, the mice fed the HFD displayed hyperlipidemia, as evidenced by increased serum levels of TC, TG, and LDL-C (*p* < 0.01) compared to the control group. However, these parameters decreased in a dose-dependent manner with the consumption of IPTV and EPTV (*p* < 0.01). Interestingly, the serum parameters in the H-IPTV and H-EPTV groups were close to those in the PC group. In the H-IPTV group, the serum TC, TG, and LDL-C levels decreased by 51.71%, 30.97%, and 54.61% (*p* < 0.01), respectively, compared to the HC group. Similarly, treatment with M-EPTV substantially reduced the serum TC, TG, and LDL-C levels by 45.74%, 41.94%, and 55.84% (*p* < 0.01) in HFD mice.

**Figure 3:**
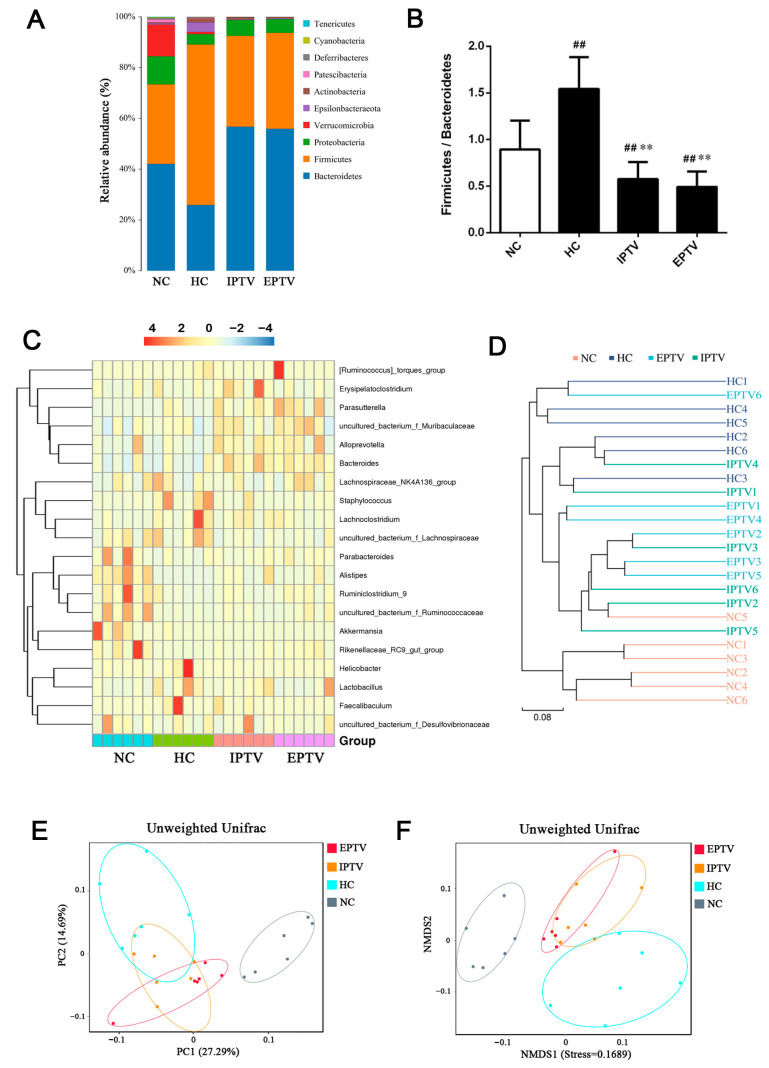
PTVs restore the high-fat-diet-induced gut bacterial dysbiosis at different taxonomic levels in HFD-fed mice. (**A**) The relative abundance of gut microbes at phylum levels; (**B**) the ratio of Firmicutes–Bacteroidetes at phylum levels; (**C**) heat map summarizing the relative abundance of gut microbes at genus levels; (**D**) evolutionary clustering analysis of bacterial community structure; (**E**) PCoA analysis of bacterial community structure; (**F**) NMDS analysis of bacterial community structure. Data are presented as means ± SD, *n* = 6. ^##^ *p* < 0.01, compared with NC group; ** *p* < 0.01, compared with HC group.

The histopathological analysis of the liver in mice from the NC, HC, PC, and dosage groups treated with IPTV and EPTV is shown in Figure 3B. It is evident that the liver tissues in the HC group mice exhibited severe pathological changes, such as significant cellular swelling, the accumulation of large fat vacuoles, and extensive nuclear disappearance. These changes indicate the presence of diffuse hepatic steatosis, inflammatory changes, and vesicular degeneration in the liver. In contrast, the normal liver cells displayed regular morphology with abundant cytoplasm, distinct nuclei, and well-defined cell borders. Notably, the treatment with IPTV and EPTV effectively mitigated lipid accumulation and fatty degeneration in a dose-dependent manner. The liver morphology and arrangement in mice treated with these two polysaccharides at a dose of 200 mg/kg bw were similar to those in normal mice. Our findings correlate to Vetter’s study, in which they found that the anti-hyperlipidemic effect of *T. versicolor* fruiting body polysaccharides was twice as potent as that of simvastatin in rats fed a high-fat diet at doses up to 200 mg/kg/d [23]. Similarly, Nikolic et al. (2023) reported a decrease in TC, TG, and LDL-C levels in hyperlipidemic rats fed with *T. versicolor* fruiting body polysaccharides for 4 consecutive weeks, particularly in the medium- and high-dose groups of *T. versicolor* polysaccharides [11]. The above results align with the observed trend of hepatic cholesterol content, indicating that PTVs effectively protect the liver against HFD-induced hyperlipidemia. 

### 3.3. PTVs Restore the High-Fat-Diet-Induced Gut Bacterial Dysbiosis at Different Taxonomic Levels in HFD-Fed Mice

Previous studies have shown a close association between alterations in the gut microbiota and abnormal lipid metabolism [24]. Dysbiosis of the gut microbiota is considered to be a vital mechanism leading to lipid metabolism disorders [25]. Polysaccharides are generally recognized as important regulators of gut flora [26]. As such, the present study sought to investigate the effects of supplementation with PTVs on the gut flora of HFD hosts. The suppressive hypolipidemic effect was evident at the highest doses of IPTV and EPTV according to the results of the serum and hepatic analyses. To determine the overall structural changes in the gut microbiota in response to PTVs at the dosage of 200 mg/kg, the 16S rRNA gene sequences of microbial samples isolated from the fecal matter of the NC, HC, IPTV, and EPTV groups were analyzed.

Initially, we conducted an examination of the similarities and disparities in the distribution of species among the NC, HC, IPTV, and EPTV groups. The extent of shared operational taxonomic units (OTUs) between these groups is summarized in the Venn diagram, which also reveals the presence of certain unique bacterial (Figure S1A) OTUs following IPTV and EPTV intervention in comparison to the HC group. Notably, both IPTV and EPTV treatment distinctly restored the HFD-induced reduction in bacterial OTU count (Figure S1B). The results of the α-diversity analysis of bacteria are presented in Figure S1C. Compared to normal mice, HFD feeding led to a decrease in the Shannon diversity and the Chao1 index, while increasing the Simpson index. The results of Zhang et al. showed the same trend [27], suggesting that HFD feeding disrupted the homeostasis of intestinal flora in normal mice. However, both IPTV and EPTV treatment distinctly enhanced their diversity (positively related to Shannon index and negatively related to Simpson index) and the richness (positively related to Chao1 index).

Next, the impact of PTVs on the abundance of main microbial phyla in the digestive tract was revealed through 16S rRNA gene sequencing data (Figure 3A). As observed in other reports [28,29], the dominant phyla were Bacteroidetes and Firmicutes in all samples. The ratio of Firmicutes to Bacteroidetes is strongly associated with the energy acquisition and conversion of the host [30]. The HC group had an elevated relative abundance of Firmicutes (by 101.55%) and a reduced abundance of Bacteroidetes (by 38.38%). In contrast, IPTV treatment was found to significantly increase in Bacteroidetes abundance (by 118.88%) and decrease the Firmicutes abundance (by 43.47%) compared to HC mice. EPTV treatment reduced the relative abundance of Firmicutes (by 115.53%) and increased the abundance of Bacteroidetes (by 40.21%) in hyperlipidemic mice. Although no consensus has been reached, a high Firmicutes–Bacteroidetes (F/B) ratio is considered an indicator of gut bacterial imbalance associated with HFDs (Figure 3B) [31,32,33]. In the present study, it was observed that HC mice had a higher ratio of Firmicutes–Bacteroidetes (*p* < 0.01), whereas IPTV and EPTV treatment resulted in a reduction in this ratio (*p* < 0.01). These findings suggest that PTVs may have the potential to counteract hyperlipidemia by modulating the Firmicutes–Bacteroidetes ratio.

**Figure 4:**
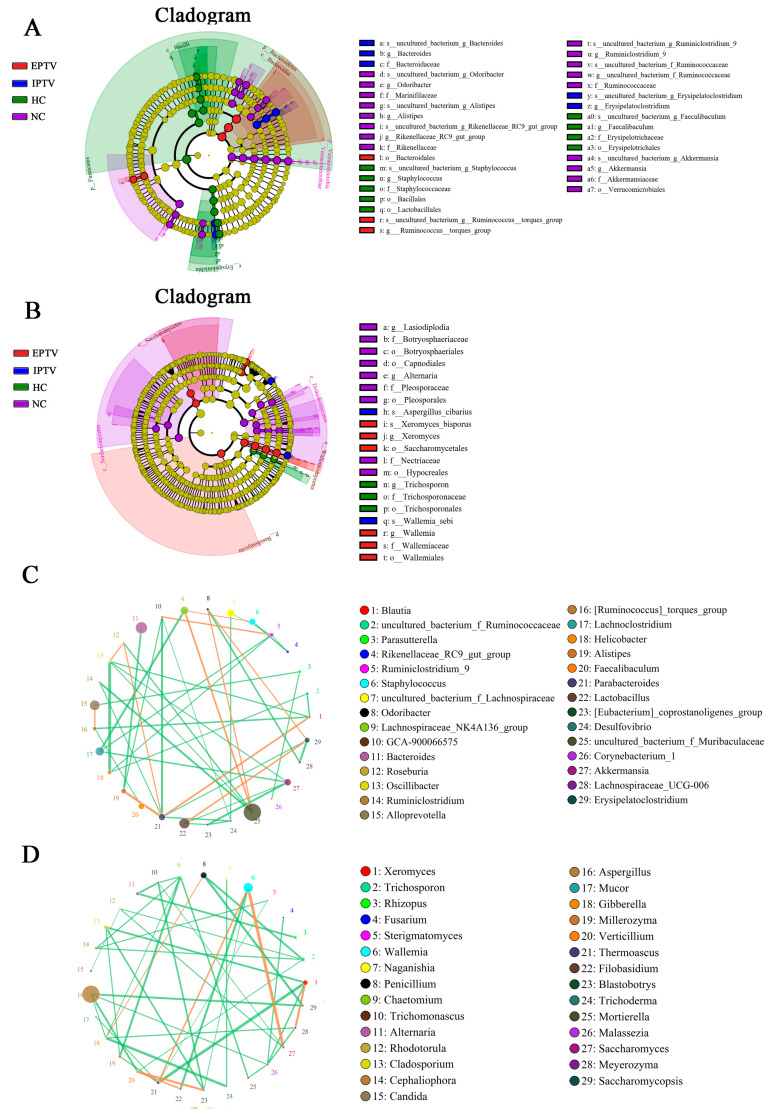
Dynamics of the intestinal microorganism in HFD-fed mice. (**A**) The cladogram of intestinal bacteria with significant differences between NC, HC, IPTV (200 mg/kg), and EPTV (200 mg/kg); (**B**) the cladogram of intestinal fungi with significant differences between NC, HC, IPTV (200 mg/kg), and EPTV (200 mg/kg); (**C**) bacterium–bacterium co-occurrence network; (**D**) fungus–fungus co-occurrence network.

Furthermore, hierarchical clustering (Figure 3C) and LEfSe analysis (Figure 4A and Figure S3A) was employed to shed light on the bacterial and fungal phenotypes that contributed to the differences in gut bacterial communities at the genus level. The abundances of the uncultured_bacterium_f_Muribaculaceae, *Lachnoclostridium*, *Lactobacillus*, uncultured_bacterium_f_Lachnospiraceae, Lachnospiraceae_ NK4A136_group, *Staphylococcus*, *Faecalibaculum*, *Helicobacter*, *Erysipelatoclostridium*, and [*Ruminococcus*]_torques_group were higher in mice fed an HFD, while the abundances of the *Alloprevotella*, *Akkermansia*, *Parabacteroides*, *Ruminiclostridium*_9, uncultured_bacterium_f_Ruminococcaceae, Rikenellaceae_RC9_gut_group, and Lachnospiraceae_UCG-006 were lower compared to the NC group. The following genera were increased in IPTV-treated mice: uncultured_bacterium_f_Muribaculaceae (by 53.45%), *Alloprevotella* (3.19-fold), *Parabacteroides* (by 29.91%), *Alistipes* (27.66-fold), uncultured_bacterium_f_Ruminococcaceae (by 19.45%), *Ruminiclostridium* (201.37%), *Odoribacter* (10.37-fold), *Oscillibacter* (37.08%), GCA-900066575 (52.74%), and Rikenellaceae_RC9_gut_group (12.74-fold) vs. the HC group. In addition, the genera *Lachnoclostridium* (by 44.18%), uncultured_bacterium_f_Lachnospiraceae (by 67.78%), Lachnospiraceae_NK4A136_group (by 38.11%), *Staphylococcus* (by 80.52%), *Helicobacter* (by 93.18%), *Desulfovibrio* (69.78%), and [*Ruminococcus*]_torques_group (by 97.02%) were decreased in IPTV-treated mice compared to HC mice. Similarly, EPTV also resulted in the increased abundance of uncultured_bacterium_f_Muribaculaceae (by 78.22%), *Alloprevotella* (2.10-fold), *Parabacteroides* (by 96.71%), *Alistipes* (14.45-fold), uncultured_bacterium_f_Ruminococcaceae (by 183.46%), *Odoribacter* (105.16-fold), *Oscillibacter* (7.89-fold), GCA-900066575 (78.97%), [*Ruminococcus*]_torques_group (by 107.73%), and Rikenellaceae_RC9_gut_group (28.58-fold). Conversely, there was a decrease in the abundance of *Lachnoclostridium* (by 67.20%), uncultured_bacterium_f_Lachnospiraceae (by 60.96%), Lachnospiraceae_NK4A136_group (by 13.47%), *Staphylococcus* (by 90.87%), *Helicobacter* (by 90.79%), *Desulfovibrio* (72.41%), and *Erysipelatoclostridium* (by 57.29%). Interestingly, the abundance of the genera *Akkermansia* and *Ruminiclostridium*_9 in EPTV-treated mice increased significantly by 492.15% and 81.17% respectively, compared to HC mice, and IPTV treatment had no noticeable effect.

To compare the beta diversity of gut microbiota among groups, PCoA and NMDS analysis based on unweighted UniFrac was carried out. As shown in Figure 3E,F, there was a clear variation in the bacterial composition of the HC group compared to the NC groups. The gut microbial community structure was significantly modified by IPTV and EPTV, with all the HFD-fed mice exhibiting a dramatic shift along the same direction, which could reverse the hyperlipidemia-induced variations in the first principal component (PC1). Evolutionary clustering analysis showed that the gut microbiota of the NC and the HC group were different, while the gut microbiota of the PTV treatment group were closer to those of the NC (Figure 3D). The results suggested that the HFD induced significant changes in the gut microbiota of mice, but the supplementation of PTVs restored them close to those of the NC group. 

To obtain a measure of microbial association, three OTU co-occurrence networks were constructed. In the bacterial co-occurrence network, OTUs assigned to unclassified groups in *Parasutterella* (OTU 3), *Lachnoclostridium* (OTU 17), *Helicobacter* (OTU 18), *Desulfvibrio* (OTU 24), and *Akkermansia* (OTU 27) were negatively correlated to other bacteria (Figure 4C). 

**Figure 5:**
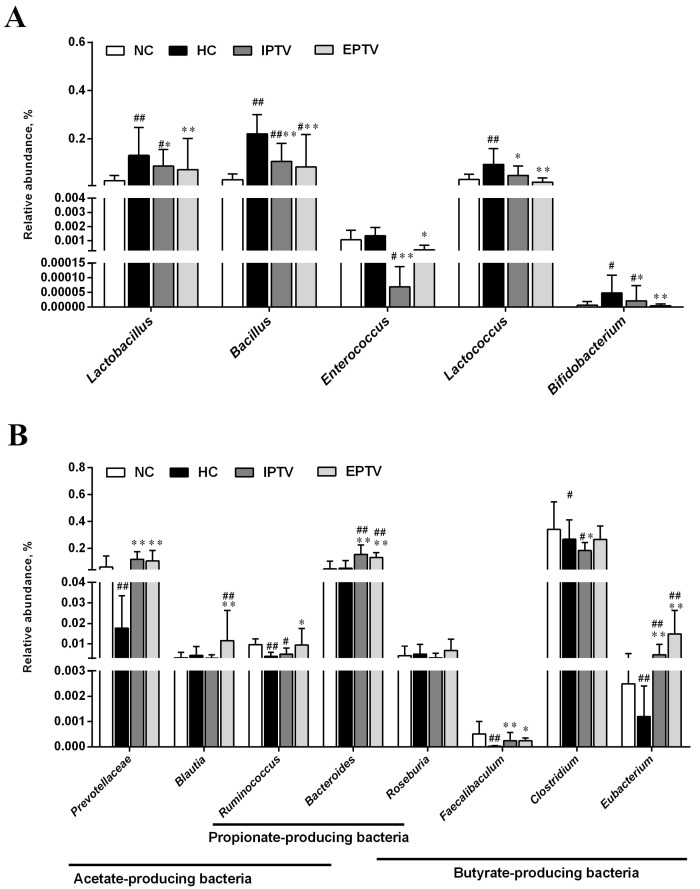
Effects of PTVs on specific gut microbial population at genus and species levels in HFD-fed mice. (**A**) The relative abundance of gut BSH-producing bacteria; (**B**) the relative abundance of gut SCFA-producing bacteria. Data are presented as means ± SD, *n* = 6. ^#^ *p* < 0.05 and ^##^ *p* < 0.01, compared with NC group; * *p* < 0.05 and ** *p* < 0.01, compared with HC group.

Regarding specific taxonomy at the genus and species level, importantly, we observed a suppression of bile-salt-hydrolase (BSH)-producing bacteria, specifically *Bacillus*, *Enterococcus*, *Bifidobacterium*, and *Lactococcus*, in mice treated with PTVs (Figure 5A). Simultaneously, there was an increase in the abundance of SCFA producers (Prevotellaceae, *Blautia*, *Bacteroides*, *Ruminococcus*, *Eubacterium*, and *Clostridium*), after PTV treatment to some extent (Figure 5B). These findings are in coincidence with previous studies. A positive correlation between the abundance of BSH-producing bacteria and cholesterol levels was observed in a study by Ou-yang et al. (2023), whereas some of the SCFA-producing bacteria were negatively correlated to high blood lipid levels [34]. According to a previous report, tempol, an antioxidant, can potently reduce *Lactococcus* and thus affect farnesoid X receptor (FARN) activity and exert anti-obesity effects [35]. Polyphenol extracts from peach peels were able to increase the relative abundance of *Bacteroides*, Prevotellaceae, and *Ruminococcus*, among others, in the intestines of mice with HFD-induced abnormal lipid metabolism [36]. Similarly, Zhou et al. found that Tartary buckwheat lowered blood lipids and upregulated the abundance of SCFA-producing bacteria such as *Blautia*, *Clostridium*, and others in the intestines of obese mice [29]. 

### 3.4. PTVs Regulated the Gut Fungal Microbes in HFD-Fed Mice

In addition to bacteria, fungi, as one of the native inhabitants of the gut, have a noteworthy role in intestinal homeostasis and host health. Although the number of intestinal fungal inhabitants is not as large as that of bacteria, more and more studies have revealed their association with host immunity, cognitive impairment, and metabolic syndrome [37,38,39]. Therefore, the present study investigated the effect of PTVs on intestinal fungi. According to the Venn diagram for fungal microbes (Figure S2A), some unique OTUs were observed after IPTV and EPTV intervention compared with the HC group. IPTV and EPTV treatment distinctly restored the HFD-induced reduction in OTU number for fungal microbes (Figure S2B). The results of the α-diversity analysis of fungi are shown in Figure S2C. IPTV and EPTV led to a slight increase in the Shannon diversity index without a significant difference, but the Simpson index decreased significantly. Meanwhile, a significantly lower Chao1 index was observed in the IPTV and EPTV groups compared with the HC group (*p* < 0.01), indicating that the richness of the gut microbiota was decreased by PTVs. 

**Figure 6:**
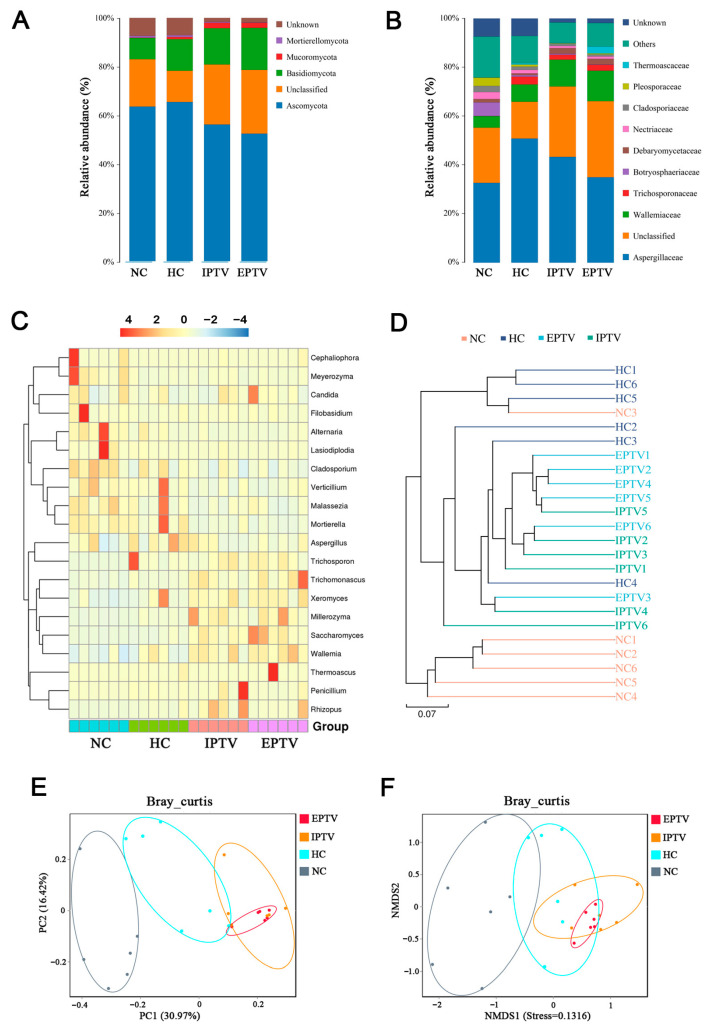
PTVs restore the high-fat-diet-induced gut fungal dysbiosis at different taxonomic levels in HFD-fed mice. (**A**) The relative abundance of gut fungal microbes at phylum levels; (**B**) the relative abundance of gut fungal microbes at family levels; (**C**) heat map summarizing the relative abundance of gut fungal microbes at genus levels; (**D**) evolutionary clustering analysis of fungal microbe community structure; (**E**) PCoA analysis of fungal microbe community structure; (**F**) NMDS analysis of fungal microbe community structure. Data are presented as means ± SD, *n* = 6.

According to ITS gene sequencing data (Figure 6A), we observed that HFD feeding was associated with a higher relative abundance of Basidiomycota (by 47.84%) and Mucoromycota (5.61-fold). After the 4-week IPTV treatment, the relative abundance of Basidiomycota and Mortierellomycota was further increased by 15.14% and 160.40%, respectively, whereas the relative abundance of Ascomycota was decreased by 14.20%. EPTV treatment also further increased the relative abundance of Basidiomycota and Mortierellomycota by 33.03% and 145.43%, respectively, and decreased the relative abundance of Ascomycota by 19.86%. A recent in vivo study in Drosophila showed that Ascomycota levels in the gut were not significantly correlated to common metabolites; however, Basidiomycota had a significant negative correlation with triglyceride levels [40]. In the human intestinal flora, the intestinal fungal group of obese individuals may be associated with an increase in Ascomycota compared to non-obese individuals [41].

In addition, hierarchical clustering and LEfSe analysis were employed to shed light on the fungal phenotypes that contributed to the differences in gut microbiota communities at the genus level (Figure 4B, Figure 6C and Figure S3). Major differences were observed at the level of dominant genus: *Aspergillus*, *Penicillium*, *Wallemia*, *Rhizopus*, *Xeromyces*, and *Trichosporon* increased, and *Lasiodiplodia*, *Cladosporium*, and *Alternaria* decreased by HFD feeding vs. the NC group. The supplementation of IPTV significantly increased the abundance of *Saccharomyces* (3.46-fold), which has the potential to ameliorate metabolic diseases [39], *Wallemia* (54.37%), *Penicillium* spp. (2.23-fold), *Millerozyma* (4.17%), *Trichoderma* spp. (1.50-fold), and *Bacillus* spp. (1.81-fold), as compared to the HC group. *Candida* spp. (22.48%) and *Rhizoctonia* spp. (4.92-fold) were also significantly more abundant, whereas the abundance of *Aspergillus* (by 31.10%)*, Lasiodiplodia* (by 37.02%)*, Cladosporium* (by 36.32%), *Trichosporon* (by 33.32%), *Thermoascus* (98.54%), *and Alternaria* (by 69.22%) were markedly decreased by supplementing with IPTV compared to the HC group. After EPTV treatment, the abundance of *Wallemia* and *Xeromyces* was increased by 32.61% and 19.61%, respectively, compared to the HC group, whereas the abundance of *Aspergillus* (by 36.00%)*, Penicillium* (by 11.58%)*, Rhizopus* (by 188.03%)*, Lasiodiplodia* (by 80.23%)*, Trichosporon* (by 20.34%), *and Alternaria* (by 56.86%) were markedly decreased by supplementing with IPTV as compared with the HC group.

Bray–Curtis-based PCoA and NMDS analyses revealed the distinct clustering of intestinal microbe communities for each experimental group (Figure 6E,F). Remarkable changes in the microbiota community structure were induced by both the HFD and PTV intervention. The gut microbiota in the HC group showed a structural change along the positive direction of PC1 compared with the NC group. The administration of IPTV further induced changes in PC1 in the positive direction to a certain extent, whereas EPTV had no effect. Furthermore, the weighted UniFrac NMDS analysis revealed that the gut bacterial communities of the IPTV and EPTV group aggregated more similarly to those of the NC group compared to the HC group. Based on the results of the evolutionary clustering analysis, the intestinal fungi between the NC and HC groups exhibited differences, while the PTV treatment group showed a trend that the intestinal fungal community was closer to the NC group (Figure 6D). This further confirmed that the HFD could change the composition of intestinal fungi in mice. However, after PTV supplementation, this change was reversed, and the intestinal fungal community returned to a level close to that of the NC. 

In the fungal network, most of the OTUs assigned to *Trichosporon* (OTU 2), *Alternaria* (OTU 11), *Cladosporium* (OTU 13), *Aspergillus* (OTU 16), *Millerozyma* (OTU 19), and *Saccharomycopsis* (OTU 29) had negative correlations with other genera, whereas the OTUs corresponding to *Wallemia* (OTU 6) co-occurred with *Thermoascus* (OTU 21) and *Saccharomyces* (OTU 27) (Figure 4D). 

Taken together, these results indicate that the consumption of PTVs induced similar gut microbe composition changes under normal conditions. 

### 3.5. Effect of PTVs on Functional Genes in HFD-Fed Mice

In order to gain a more fundamental understanding of the altered gut microbiota, we predicted their functional profiles using Phylogenetic Investigation of Communities by Reconstruction of Unobserved States (PICRUSt) analysis. 

**Figure 7:**
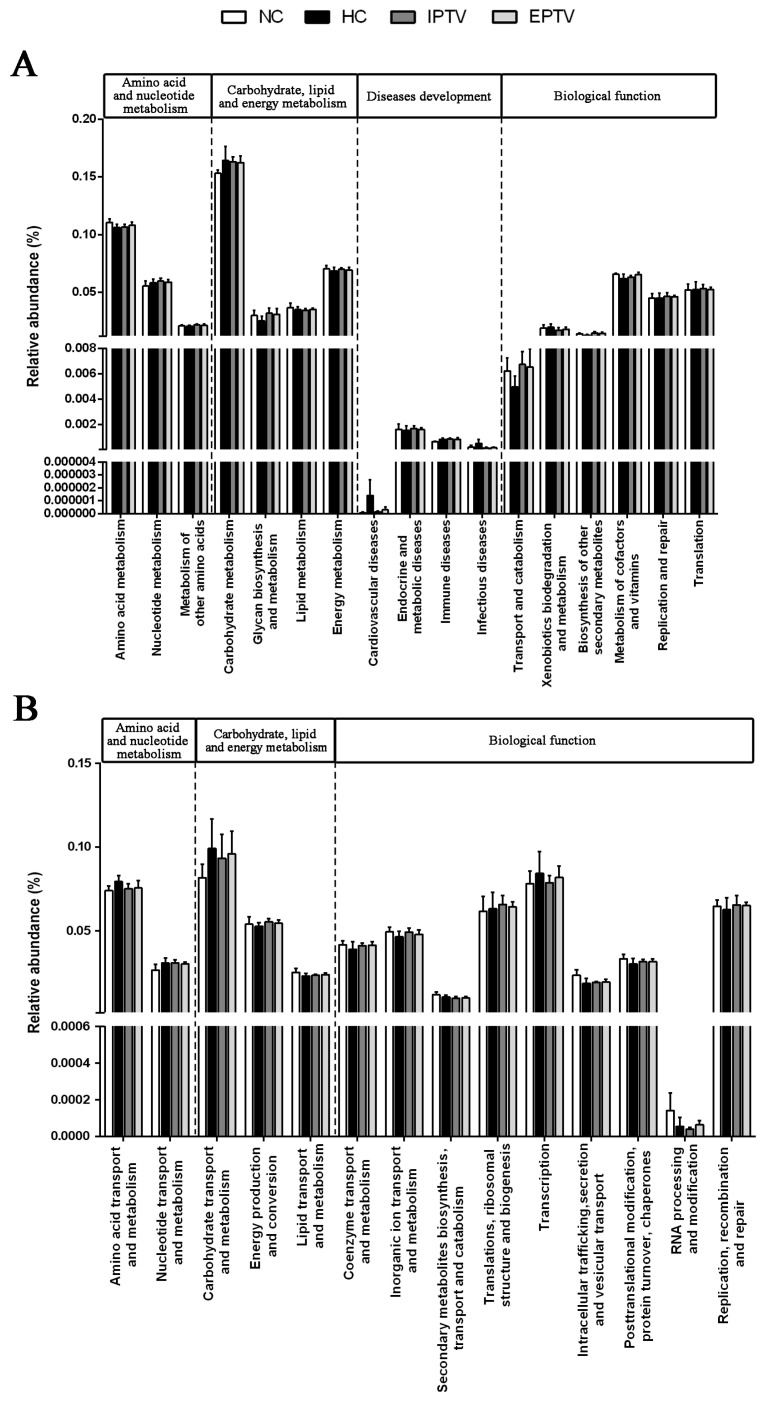
Prediction of functional pathway using PICRUSt analysis. (**A**) Total KEGG pathways changed in all groups. (**B**) COG pathways changed in all groups.

As shown in Figure 7A and Figure S4, for the Kyoto Encyclopedia of Genes and Genomes (KEGG) pathways based on the composition of gut microbiota compared with the NC group, HFD feeding led to different changes in these pathways, including amino acid and nucleotide metabolism (amino acid metabolism increased to 0.1061 vs. 0.1106), carbohydrate, lipid metabolism (carbohydrate metabolism increased to 0.1718 vs. 0.1546; energy metabolism decreased to 0.0667 vs. 0.0691; glycan biosynthesis and metabolism decreased to 0.0233 vs. 0.0276), disease development (cardiovascular diseases increased to 5.68 × 10^−5^ vs. 2.08 × 10^−5^; immune diseases increased to 8.44 × 10^−4^ vs. 6.25 × 10^−8^), and other biological functions (biosynthesis of other secondary metabolites decreased to 0.0126 vs. 0.0139; transport and catabolism decreased to 0.0049 vs. 0.0057). To various degrees, both IPTV and EPTV treatment could alleviate the change in the relative abundance of gut microbiota induced by HFD. Of note, supplementation with PTVs led to a distinct reduction in bacterial populations involved in cardiovascular diseases in HFD-fed mice. A similar trend was observed in the Clusters of Orthologous Groups of proteins (COG) pathway analysis (Figure 7B and Figure S5). Both IPTV and EPTV treatment, to some extent, restored the HFD-induced modification of bacterial populations involved in energy metabolism, biological structures, and other above biological functions, mitigating the damage caused by the HFD.

### 3.6. PTVs Upregulate Butyrate Production by Buk and Butyryl-CoA Enzymes Accompanied by an Increase in SCFA Receptors

**Figure 8:**
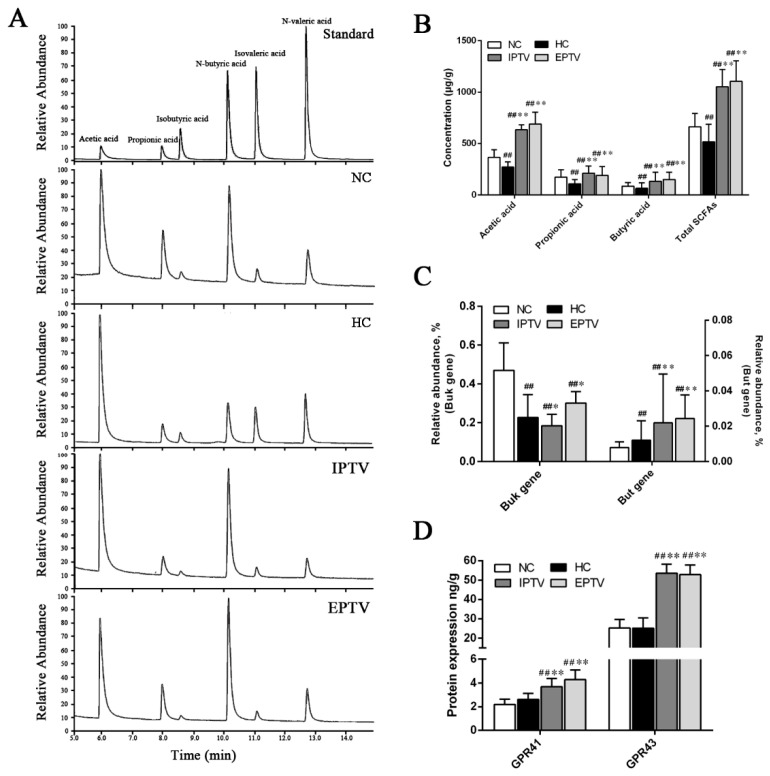
PTVs upregulates butyrate production by *buk* and *but* genes accompanied by an increase in SCFA receptor expression in HFD-fed mice. (**A**) GC–MS chromatograms of SCFA in feces. (**B**) SCFA concentration in feces measured by GC–MS. (**C**) Predominant butyrate-producing genes: relative abundance of butyrate kinase (*buk*) and butyryl-CoA (*but*) bacterial genes in feces. (**D**) Protein expression of SCFA receptors (GPR41, GPR43) in colon. Data are presented as means ± SD, *n* = 5. ^##^ *p* < 0.01, compared with NC group; * *p* < 0.05 and ** *p* < 0.01, compared with HC group.

Dietary interventions are important for butyrate synthesis in the ileum, and the synthesis of SCFAs, especially butyrate, is associated with multiple metabolic beneficial effects [42]. GC–MS analyses revealed that PTVs restored SCFA production, which was reduced in response to hyperlipidemia (Figure 8A). The HFD caused a significant decrease in SCFAs in the intestines of mice compared with the normal diet (*p* < 0.01). Compared with the hyperlipidemic control, acetic acid, propionic acid, and butyric acid were increased by 133.42% (*p* < 0.01), 95.92%, and 105.94% (*p* < 0.01) in the IPTV group, respectively, while supplementation with EPTV increased intestinal acetic acid, propionic acid, and butyric acid by 154.02% (*p* < 0.01), 76.98% (*p* < 0.01), and 130.31% (*p* < 0.01) in HFD-fed mice, respectively (Figure 8B). The contents of isobutyric acid and valeric acid were elevated after undergoing IPTV and EPTV treatment (Figure S6). Therefore, the content of total SCFAs was increased after both IPTV and EPTV intervention. 

Typically, two genes, butyrate kinase (*buk*) and acetate CoA-transferase (butyryl-CoA, *but*), are used as biomarkers for the detection of butyrate-producing communities [43]. We hypothesized that HFD and PTV treatment could affect the growth of specific commensal species like *Faecalibacterum* (involved in the *but* pathway), *Roseburia* (involved in the *but* pathway), *Eubacterium* (involved the in *but* pathway), and *Clostridium* (involved in the *buk* pathway), which are heavy producers of butyrate. Compared with the NC group, the average counts of *Faecalibacterum* in stool samples from HC mice were significantly increased (*p* < 0.05, Figure 5B), but the average counts of *Eubacterium* and *Clostridium* were significantly decreased (*p* < 0.01). Therefore, the HFD only inhibited the *buk* gene but not the *but* gene, suggesting that HFD inhibited butyrate via the *buk* pathway. However, IPTV administration significantly increased bacteria carrying the *but* gene (by 80.95%, *p* < 0.01) by increasing the relative abundance of *Eubacterium* (Figure 5B), but decreased bacteria carrying the *buk* gene (18.87%) by decreasing the relative abundance of *Clostridium*. Notably, bacteria carrying the *buk* (by 101.48%, *p* < 0.01) and *but* (by 33.14%, *p* < 0.01) genes were elevated by increasing the relative abundance of *Eubacterium, Roseburia*, and *Clostridium* in the EPTV group, respectively, vs. the hyperlipidemic control, demonstrating that PTVs could promote the growth of butyrate-producing bacteria (Figure 8C). 

SCFAs conferred some of their biological effects via the G-protein-coupled receptors known as GPR43 and GPR41. Much attention has been paid to the beneficial roles of GPR43 in energy and glucose homeostasis [44,45]. Given the effects on SCFA production of PTVs, we measured the expression of SCFA receptors by ELISA. The protein masses of SCFA receptors GPR41 and GPR43 were increased significantly in the colons of IPTV-treated mice by 40.52% and 113.24% (*p* < 0.01), respectively, and increased in the colons of EPTV-treated mice by 62.42% and 110.28% (*p* < 0.01), respectively (Figure 8D).

Together, these data suggest that the PTVs, as a prebiotic, promote the growth of intestinal butyrate-producing bacteria by the *buk* and *but* pathways, accompanied by an increase in SCFAs (especially butyrate) and their receptors. 

### 3.7. Intestinal Flora Correlate with Metabolic Parameters

**Figure 9:**
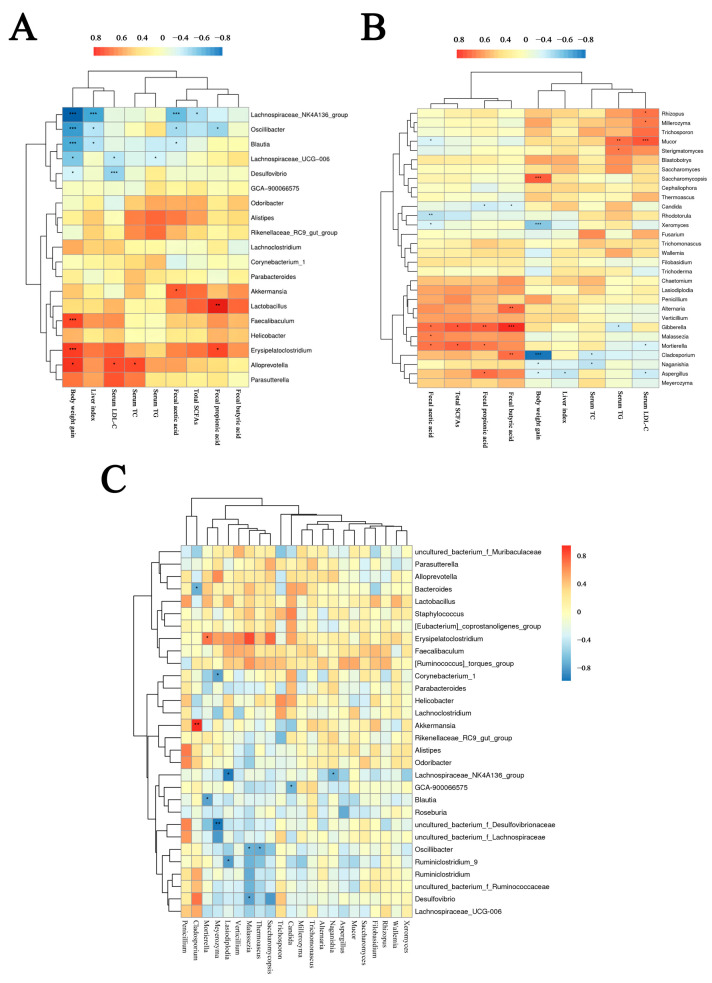
Spearman’s correlations between the intestinal microbe and lipid metabolic parameters. (**A**) Heat map describing Spearman’s correlations between the intestinal bacterial community and lipid metabolic parameters. (**B**) Heat map describing Spearman’s correlations between the intestinal fungal community and lipid metabolic parameters. (**C**) Heat map describing Spearman’s correlations between the intestinal bacterial and fungal communities. * *p* < 0.05, ** *p* < 0.01, *** *p* < 0.001.

To further elucidate the specific intestinal flora that may ameliorate the genus levels of HFD-induced obesity, the correlations between the metabolic parameters (body weight, liver weight, serum lipid level, hepatic lipid level, lipid metabolic enzyme, and fecal SCFAs levels) and the gut microbiota (the relative abundances of top 30 key genera) associated with lipid metabolism were calculated using Spearman’s analysis (Figure 9). Regarding bacteria, the HC group enriched in *Erysipelatoclostridium* and *Faecalibaculum* was positively correlated to body weight gain. The PTV-enriched *Oscillibacter* exhibited negative relationships with body weight gain and liver index, while Lachonospiraceae_UCG-006 were negatively related to body weight gain and serum LDL-C level. *Akkermansia* was positively correlated to the fecal acetic acid content. Likewise, a recent study has shown that *Erysipelatoclostridium* has a role in mediating the exacerbation of obesity [46]. Increases in *Faecalibaculum* tend to be accompanied by an increase in body weight, whereas *Oscillibacter* exhibits the opposite trend [47]. In addition, *Akkermansia* is considered a probiotic that strengthens the intestinal barrier and has the potential to alleviate obesity-related metabolic syndrome [48]. The HC group that was enriched in *Mucor* and *Trichosporon* exhibited positive relationships with serum lipid profiles. Of note, the network of genera of 16S RNA genes and ITS sequences revealed a strong relationship between bacteria and fungi. The relative abundance of the Lachnospiraceae_NK4A136_group enriched in PTVs was negatively related to *Naganishia* and *Lasiodoplodia.* The PTV-enriched *Oscillibacter* was negatively related to *Malassezia* and *Thermoascus*. 

Different polysaccharides generate various results, which may be attributed to their diverse structures and multiple hydrolysis mechanisms. After the degradation of glycoside by specific polysaccharides-degrading microbes, the free monosaccharides act as carbon sources and energy for proliferation or SCFA production [49]. SCFAs have a variety of physiological functions, including anti-inflammatory functions and inhibiting the growth of pathogenic microorganisms [50]. Bacteria and fungi coexist in the intestines, and it has been reported that a high-fat diet alters the structure of intestinal fungal and bacterial communities, affecting the interactions and functions of intestinal microbial communities [51]. The interactions and synergistic effects between intestinal fungi and bacteria are complex [52], and the mechanisms by which PTVs regulate such interactions deserve further investigation.

## 4. Conclusions

In summary, the results of this study indicated that PTVs ameliorated hepatic fat accumulation and normalized the dysbiosis of intestinal fungal and bacterial communities in HFD states. In addition, PTVs could enhance the presence of bacteria that produce SCFAs and activate SCFA receptors, leading to increased levels of SCFAs, particularly butyric acid. PTVs also decreased the relative abundance of BSH-producing bacteria, which may inhibit the activity of intestinal BSH enzymes and improve liver and serum cholesterol levels. These findings suggest that PTVs may be a promising dietary functional food component for preventing weight gain and obesity-related metabolic syndrome. 

## Data Availability

The data presented in this study can be made available by the corresponding author upon request.

## Supplementary Materials

The following supporting information can be downloaded at: https://www.mdpi.com/article/10.3390/microorganisms12081654/s1, Table S1: Composition of diets fed to Kunming mice in animal experiment; Table S2: Effects of IPTV and EPTV on body weight and organ index in HFD mice; Figure S1: PTVs modulate the composition of gut microbiota in HFD-fed mice; Figure S2: PTVs modulate the composition of intestinal fungi in HFD-fed mice; Figure S3: LDA scores of (A) gut bacteria and (B) intestinal fungi in mice of different treatment groups; Figure S4: KEGG functional prediction analysis of (A) gut microbiota and (B) intestinal fungi in various groups of mice; Figure S5: COG functional prediction analysis of (A) gut microbiota and (B) intestinal fungi in various groups of mice; Figure S6: Concentration of SCFAs in feces of different treatment groups.
