# Supplementary material for: Polysaccharides from Trametes versicolor as a Potential Prebiotic to Improve the Gut Microbiota in High-Fat Diet Mice
Source: Microorganisms. 2024 Aug 13;12(8):1654. doi: 10.3390/microorganisms12081654 (PMC11356736; doi:10.3390/microorganisms12081654)
Supplement: Supplementary file 1 [file microorganisms-12-01654-s001.zip › microorganisms-3118390-supplementary.pdf]

Table S1 Composition of diets fed to Kunming mice in animal experiment.

	Standard diet	High-fat diet
Protein (kcal %)	21	18.7
Carbohydrate (kcal %)	67	59.6
Fat (kcal %)	12	21.7
Ingredient, g/kg		
Casein	140	124.2
Corn starch	391.2	347
Maltodextrin	150	133.1
Cellulose	50	44.4
Soybean oil	13.3	11.8
Starch	184.7	163.8
Calcium hydrophosphate	15	13.3
Calcium carbonate	5	4.4
Amino acid mixture	2.8	2.5
Mineral mixture	35	31
Vitamin mixture	10	8.8
Cholesterol	0	10
Lard	0	100
Sodium cholate	3	5.7
Total	1000	1000

Table S2 Effects of IPTV and EPTV on body weight and organ index in HFD mice.

Groups	Body weight			Organ index			
	Initial (g)	Final (g)	Body weight gain (g)	Liver index (%)	Spleen index (%)	Heart index (%)	Kidney index (%)
NC	24.60±3.11	37.51±2.03	15.83+3.07	4.01±0.44	0.27±0.04	0.53±0.04	1.36±0.14
HC	25.40±1.05	43.47±3.30	18.43+2.55	5.89±0.37##	0.26±0.07	0.54±0.08	1.36±0.14
PC	24.52±3.42	43.30±2.10	19.02+2.81	4.50±0.36**	0.24±0.05	0.41±0.04	1.36±0.14
L-IPTV	24.44±4.67	43.65±3.43	20.37+3.31	4.91±0.69*	0.27±0.05	0.44±0.05	1.36±0.14
M-IPTV	24.05±0.95	43.98±1.43	17.96+2.47	4.79±0.34*	0.27±0.04	0.49±0.10	1.36±0.14
H-IPTV	24.86±1.36	43.76±2.40	18.91+3.75	4.29±0.28**	0.27±0.03	0.50±0.06	1.36±0.14
L-EPTV	24.40±0.94	42.14±4.03	15.89+4.34	4.74±0.45*	0.26±0.04	0.51±0.09	1.36±0.14
M-EPTV	23.94±0.91	43.86±1.63	18.62+2.56	4.66±0.30*	0.28±0.04	0.47±0.09	1.36±0.14
H-EPTV	24.25±0.58	42.24±3.31	18.65+5.54	4.59±0.70**	0.26±0.05	0.45±0.06	1.36±0.14

Abbreviation: NC, normal control; HC, hyperlipidemic control; PC, positive control; IPTV, intracellular polysaccharides of *Trametes versicolor*; EPTV, extracellular polysaccharides of *Trametes versicolor*. Data are presented as means ± SD, n = 10. $^{\#}p < 0.05$, compared with NC group. **$p < 0.01$, compared with HC group.

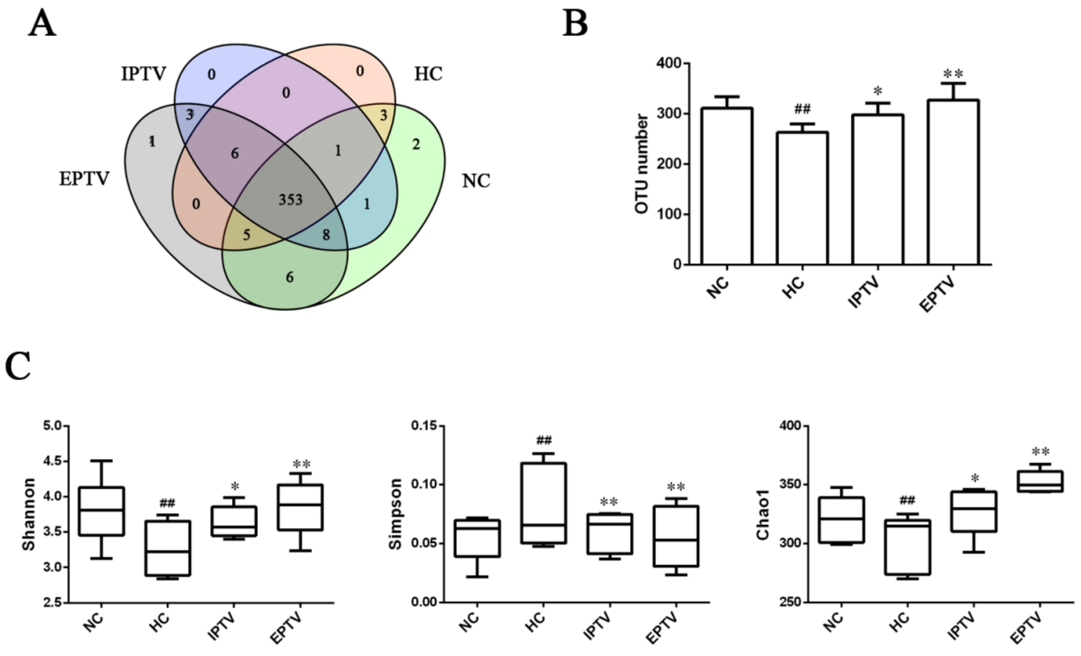

Figure S1 PTVs modulates the composition of gut microbiota in HFD fed mice. (**A**) Venn analysis and (**B**) OTU number in different groups. The diversity of gut microbiota was estimated by (**C**) Shannon, Simpson, and Chao1 indexes. $^{\#}p < 0.05$ and $^{\#\#}p < 0.01$, compared with NC group; * $p < 0.05$ and ** $p < 0.01$, compared with HC group.

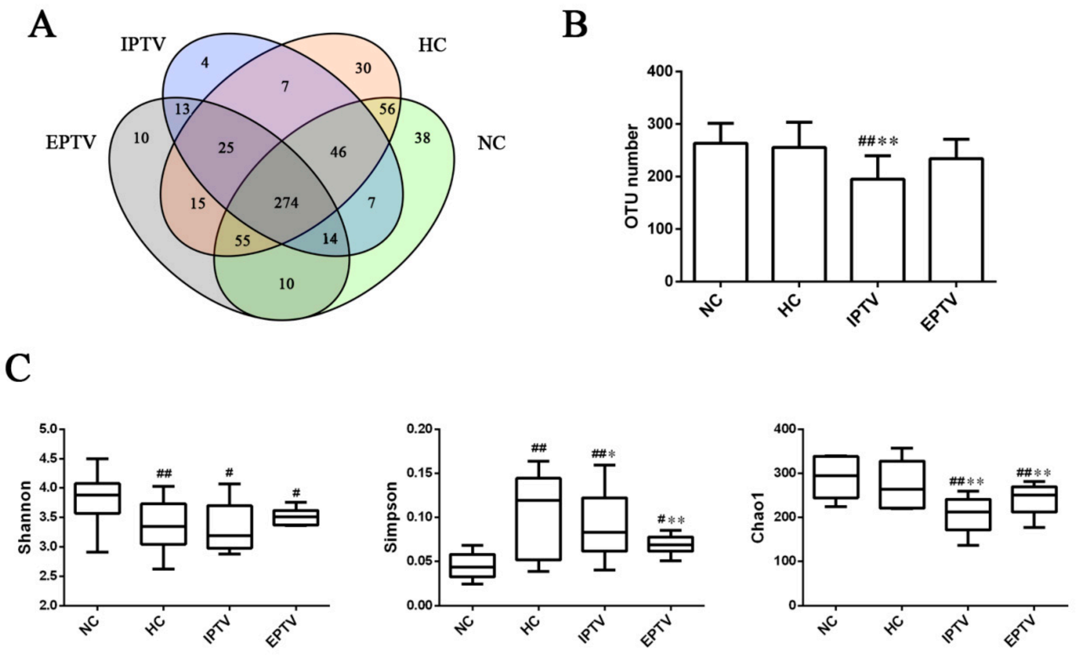

Figure S2 PTVs modulates the composition of intestinal fungi in HFD fed mice. (**A**) Venn analysis and (**B**) OTU number in different groups. The diversity of intestinal fungi was estimated by (**C**) Shannon, Simpson, and Chao1 indexes. $^{\#}p < 0.05$ and $^{\#\#}p < 0.01$, compared with NC group; * $p < 0.05$ and ** $p < 0.01$, compared with HC group.

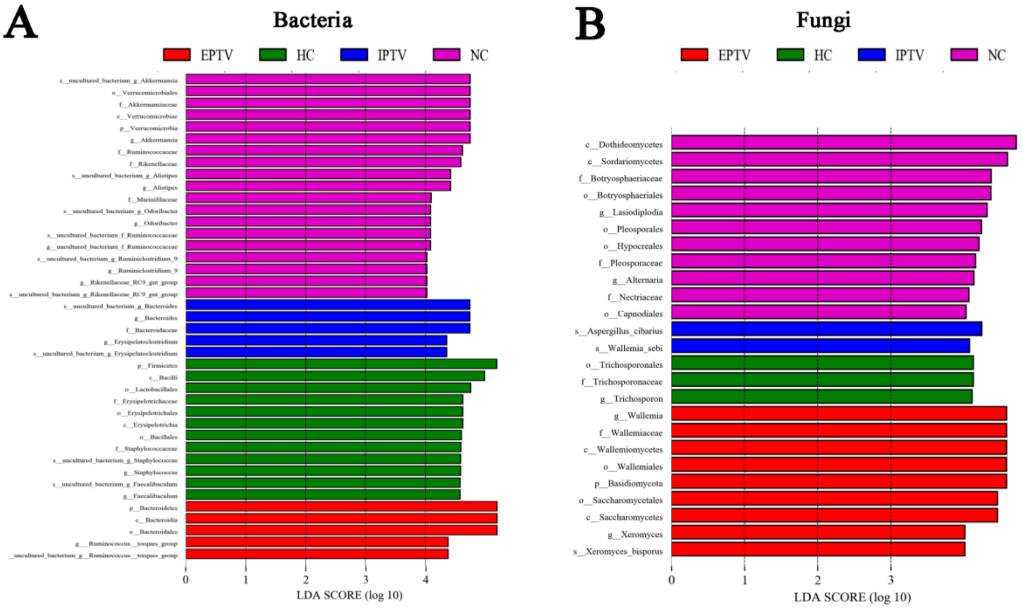

Figure S3 LDA scores of (**A**) gut bacteria and (**B**) intestinal fungi in mice of different treatment groups.

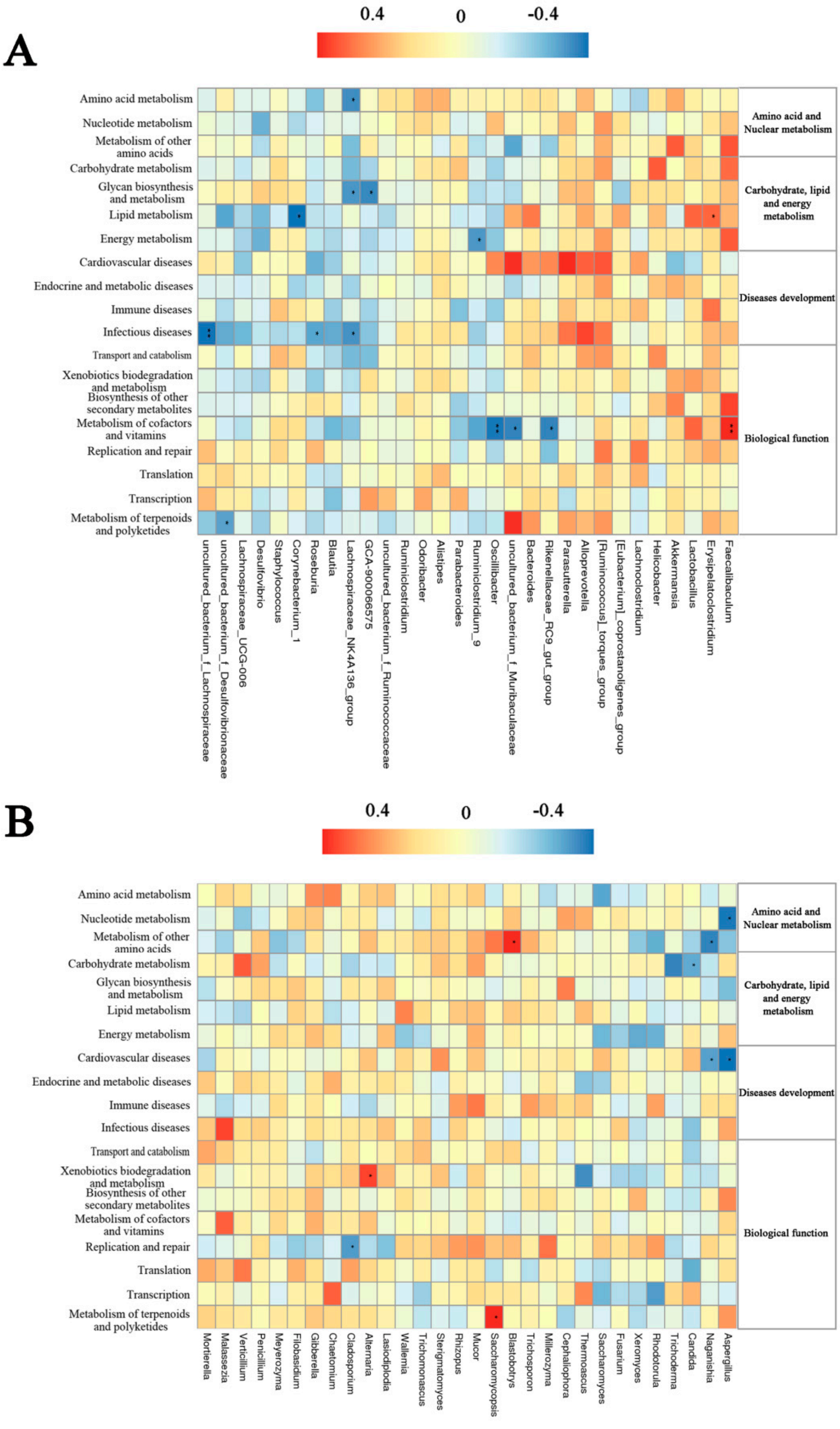

Figure S4 KEGG functional prediction analysis of (**A**) gut microbiota and (**B**) intestinal fungi in various groups of mice.

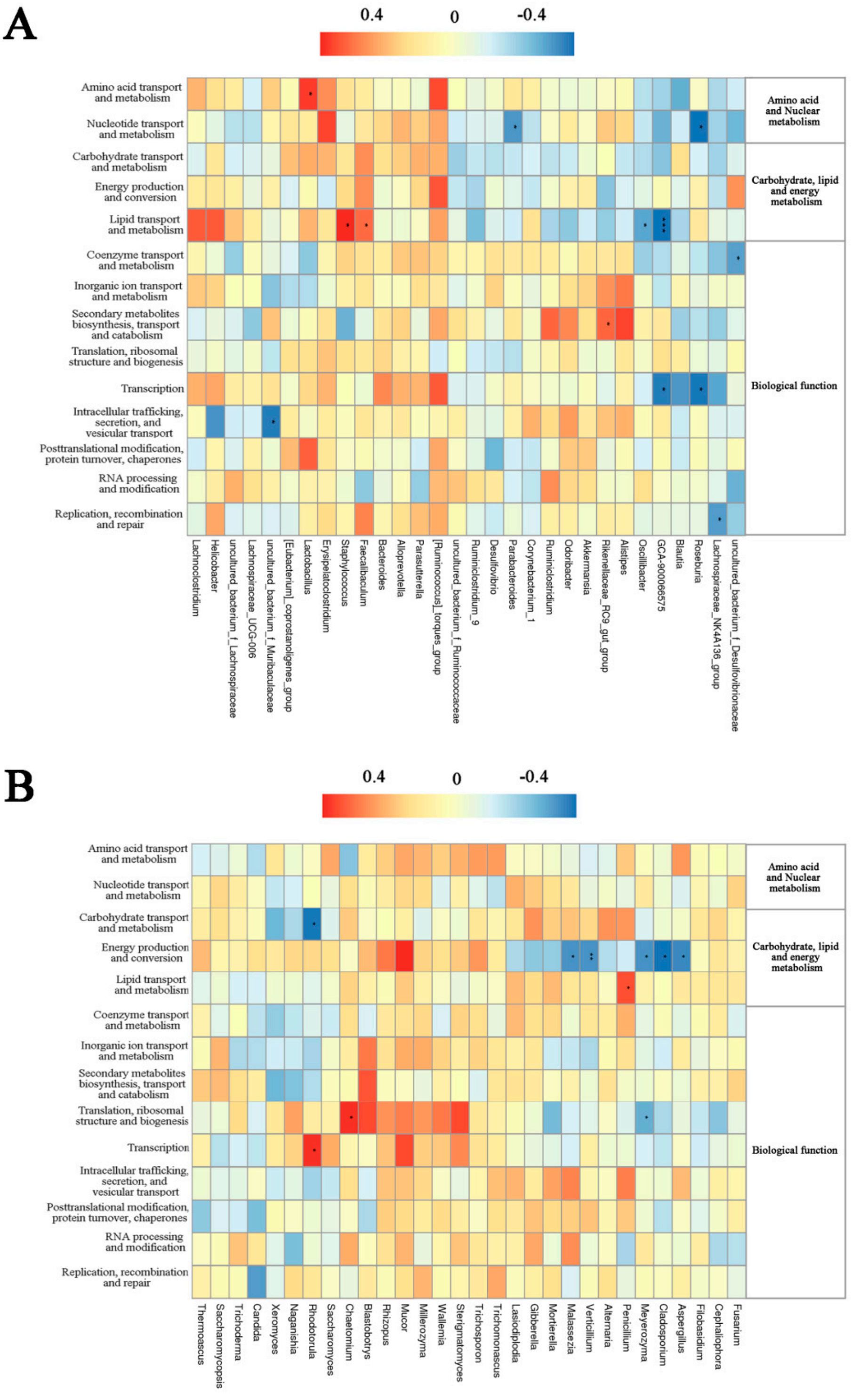

Figure S5 COG functional prediction analysis of (**A**) gut microbiota and (**B**) intestinal fungi in various groups of mice.

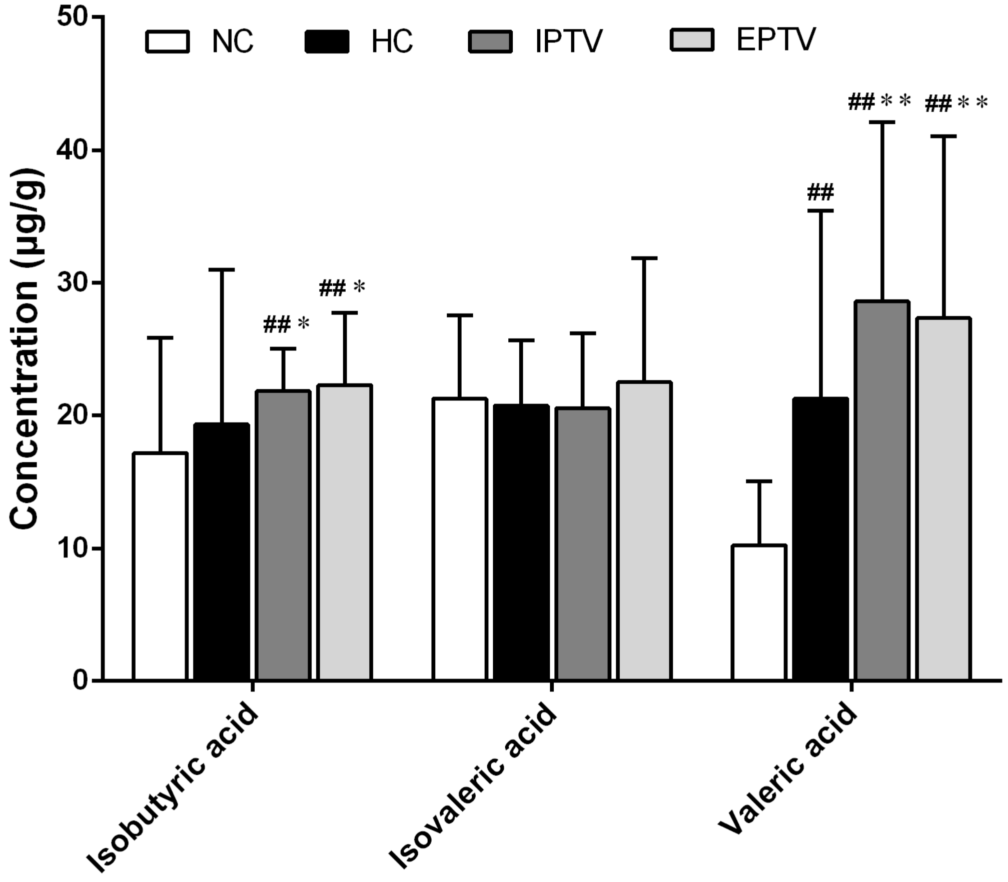

Figure S6 Concentration of SCFAs in feces of different treatment groups.
